# Forming cooperative representations via solipsistic synaptic plasticity rules

**DOI:** 10.1186/1471-2202-12-S1-P69

**Published:** 2011-07-18

**Authors:** Joel Zylberberg, Michael R DeWeese

**Affiliations:** 1 Department of Physics, University of California, Berkeley, CA 94720, USA; 2 Redwood Center for Theoretical Neuroscience, University of California, Berkeley, CA 94720, USA; 3 Helen Wills Neuroscience Institute, University of California, Berkeley, CA 94720, USA

## 

The canonical model of primary visual cortex (V1) is that it forms a linear generative model of the image stimulus presented to the eyes. Thus, for a given image with pixel values X_j_, the representation X_j_*=∑_i_b_i_ψ_ij_ is formed by multiplying the activity of each neuron (b_i_) by the feature that neuron encodes (ψ_ij_), and summing over all neurons. We call this a *cooperative* representation, since it involves all of the neurons collectively forming a single representation. Over time, the network is thought to adapt so as to minimize, on average, the mean-squared error between the representation X* and the input X, ||X-X*||^2^. Performing gradient descent on this error function yields the usual learning rule Δψ_ij_= α b_i_(X_j_- ∑_i_b_i_ψ_ij_ ), where α is some small positive constant called the learning rate. Typically, the features ψ_ij_ are interpreted as the receptive fields (RF’s; features to which a neuron responds) of the neurons; indeed, there is strong evidence [1] that the feature encoded by a neuron is very similar to its RF. In that case, the value ψ_ij_ can be thought of as the strength of the synaptic connection between input pixel value X_j_, and neuron i. With that interpretation in mind, it is clear that the canonical learning rule Δψ_ij_= α b_i_(X_j_- ∑_i_b_i_ψ_ij_ ), used by most previous work in this field [1,2], fails to be biologically realistic because the rule for updating one synaptic strength ψ_ij_ requires knowledge of the strengths of many synaptic connections, all on different neurons (with indices i), and it is not clear that such information is available to each individual synapse in the brain.

We consider instead a Hebbian learning rule that respects synaptic locality, Δψ_ij_= α b_i_(X_j_- b_i_ψ_ij_ ) [3]. In this case, the information required to change the strength of synapse ψ_ij_ consists solely of the pre-synaptic activity X_j_, the post-synaptic activity b_i_, and the current strength of the synaptic connection ψ_ij_. While this rule respects the locality of synaptic information, it does not appear to perform gradient descent on the desired error function ||X_j_- ∑_i_b_i_ψ_ij_||^2^. Instead, our local rule can be seen as gradient descent on the error function ∑_i_||X_j_- b_i_ψ_ij_ ||^2^, which is the sum over all neurons of the error between each neuron’s own internal representation of the input, b_i_ψ_ij_, and the input image. In other words, a network that follows Oja’s [3] local learning rule is a *solipsistic* one: each neuron makes its own individual representation of the input, and learning optimizes each of those representations individually.

We have proven that, if neuronal activities {b_i_} are uncorrelated, and sufficiently sparse (the majority of the b_i_’s are zero for any given image), the local and non-local learning rules are approximately equal, when averaged over many image presentations: <Δψ_ij_> = α <b_i_(X_j_- b_i_ψ_ij_)> ≈ α <b_i_(X_j_- ∑_i_b_i_ψ_ij_ )>. This suggests a previously undiscovered role for independence and sparseness in visual cortex: these properties allow the neuronal network to (approximately) form the optimal cooperative representation, despite the locality of its learning rules. The same proof applies other neuronal networks that form linear generative models.

We will present the details of our proof, and an example network (similar to that of [4]) of leaky integrate-and-fire neurons that learns a sparse image code using the local learning rule Δψ_ij_= α b_i_(X_j_- b_i_ψ_ij_ ). In our network, inhibitory inter-neuronal connections and variable firing thresholds keep the neuronal activities uncorrelated and sparse throughout the learning process. When trained on natural scenes, this network learns the same diversity of receptive fields as do previous non-local algorithms [1,2].

## References

[B1] Olshausen BA Field DJ Emergence of simple cell-receptive fields by learning a sparse code for natural images Nature 1996 381 607 609 10.1038/381607a0 8637596

[B2] Rehn M Sommer FT A network that uses few active neurones to code visual input predicts the diverse shapes of cortical receptive fields J Comput Neurosci 2007 22 135 146 10.1007/s10827-006-0003-9 17053994

[B3] Oja E A simplified neuron model as a principal component analyzer J Math Biol 1982 15 267 273 10.1007/BF00275687 7153672

[B4] Falconbridge MS Stamps RL Badcock DR A simple Hebbian/anti-Hebbian network learns the sparse, independent components of natural images Neural Comput 2006 18 415 429 10.1162/089976606775093891 16378520

